# Neuronal network mechanisms associated with depressive symptom improvement following electroconvulsive therapy

**DOI:** 10.1017/S0033291720001518

**Published:** 2021-12

**Authors:** Akihiro Takamiya, Taishiro Kishimoto, Jinichi Hirano, Shiro Nishikata, Kyosuke Sawada, Shunya Kurokawa, Bun Yamagata, Toshiaki Kikuchi, Masaru Mimura

**Affiliations:** 1 Department of Neuropsychiatry, Keio University School of Medicine, Tokyo 160-8582, Japan; 2 Center for Psychiatry and Behavioral Science, Tokyo 193-8505, Japan

**Keywords:** electroconvulsive therapy, functional connectivity, functional magnetic resonance imaging, hippocampus, multivoxel pattern analysis

## Abstract

**Background:**

Electroconvulsive therapy (ECT) is the most effective antidepressant treatment for severe depression. Although recent structural magnetic resonance imaging (MRI) studies have consistently reported ECT-induced hippocampal volume increases, most studies did not find the association of the hippocampal volume changes with clinical improvement. To understand the underlying mechanisms of ECT action, we aimed to identify the longitudinal effects of ECT on hippocampal functional connectivity (FC) and their associations with clinical improvement.

**Methods:**

Resting-state functional MRI was acquired before and after bilateral ECT in 27 depressed individuals. *A priori* hippocampal seed-based FC analysis and a data-driven multivoxel pattern analysis (MVPA) were conducted to investigate FC changes associated with clinical improvement. The statistical threshold was set at cluster-level false discovery rate-corrected *p* < 0.05.

**Results:**

Depressive symptom improvement after ECT was positively associated with the change in the right hippocampus-ventromedial prefrontal cortex FC, and negatively associated with the right hippocampus-superior frontal gyrus FC. MVPA confirmed the results of hippocampal seed-based analyses and identified the following additional clusters associated with clinical improvement following ECT: the thalamus, the sensorimotor cortex, and the precuneus.

**Conclusions:**

ECT-induced change in the right frontotemporal connectivity and thalamocortical connectivity, and changes in the nodes of the default mode network were associated with clinical improvement. Modulation of these networks may explain the underlying mechanisms by which ECT exert its potent and rapid antidepressant effect.

## Introduction

Neuroimaging studies have revealed that neuronal network disorganization is related to various depressive symptoms (Northoff, 2016), supporting the notion of depression as a neuronal network disorder (Mayberg, 2009; Price & Drevets, 2010). Indeed, abnormal resting-state functional connectivity (FC) among certain brain regions has been reported in depressed individuals: those brain regions include the subcallosal cingulate cortex (SCC), thalamus, medial prefrontal cortex (MPFC), orbitofrontal cortex (OFC), medial temporal lobe (MTL), and precuneus (Cheng et al., 2016; Greicius et al., 2007; Kaiser, Andrews-Hanna, Wager, & Pizzagalli, 2015). Antidepressant medications (Meyer et al., 2019; Wang et al., 2019), cognitive behavioral therapy (CBT) (Rubin-Falcone et al., 2020), and transcranial magnetic stimulation (TMS) (Philip et al., 2018) had various effects on FC among distributed neuronal networks. Among several antidepressant treatments, electroconvulsive therapy (ECT) is the most effective and rapidly acting treatment (Kellner et al., 2012; Spaans et al., 2015). Although the underlying mechanisms of ECT action are still unclear, studying the mechanisms by which ECT exerts its potent and rapid antidepressant effect, as well as its cognitive side effect, could advance our ability to develop precisely targeted antidepressant treatments that are fast-acting and effective with less side effects (McMullen & Lisanby, 2019).

One of the most consistent brain regions that have been reported in ECT literature so far is the hippocampus. A recent meta-analysis (Takamiya et al., 2018) and mega-analysis (Oltedal et al., 2018) showed ECT-related hippocampal volume increase. However, the clinical relevance of this hippocampal volume increase is unclear. The degree of the increased volume of the whole hippocampus did not correlate with improvement of depressive symptoms (Oltedal et al., 2018; Sartorius et al., 2019; Takamiya et al., 2018), whereas volume changes in a specific hippocampal subfield (e.g. dentate gyrus) correlated with clinical improvement (Nuninga et al., 2019; Takamiya et al., 2019a, 2019b). Similar to this evidence, the relationship between hippocampal volume change and cognitive function is also inconsistent (Abbott et al., 2014; Nordanskog, Larsson, Larsson, & Johanson, 2014; Van Oostrom et al., 2018).

Since the hippocampus has direct connections with several brain regions implicated in depression, such as the amygdala and ventromedial prefrontal cortex (vmPFC) (Poppenk, Evensmoen, Moscovitch, & Nadel, 2013), investigating the effect of ECT on the hippocampal network may be beneficial. Moreover, preclinical studies have shown that electroconvulsive stimulation (ECS), an animal model of ECT, induced synaptogenesis and regulated key molecules for synaptic plasticity in the hippocampus (Chen, Madsen, Wegener, & Nyengaard, 2009; Nordgren et al., 2013), which could lead to the reorganization of the human brain's neuronal network. Abbott et al., reported that within a geriatric depressed cohort (mean 65.3 years old) that changes in the right hippocampal FC with the right temporal lobe correlated with symptom improvement, but not with cognitive performance (Abbott et al., 2014). Bai et al., reported that within a middle-aged cohort (mean 38.0 years old), changes in the left anterior hippocampal FC with the right medial temporal gyrus (MTG) correlated with changes in depressive symptoms, and changes in the left posterior hippocampal FC with the bilateral angular gyrus (AG) correlated with changes in verbal fluency scores (Bai et al., 2019). Compared with findings of the effect of ECT on hippocampal volumes, our understanding of the effect of ECT on the hippocampal network is limited.

Based on previous findings regarding the close relationships between depression, the hippocampus, and ECT, we hypothesized that the clinical effect of ECT was mediated by hippocampal network change. To test this hypothesis in the current study, we acquired resting-state functional magnetic resonance imaging (rs-fMRI) data before and after a course of ECT, and we performed hippocampal seed-based FC analyses. We also analyzed whole-brain FC with multivoxel pattern analysis (MVPA) to validate our hypothesis-driven analyses and to investigate FC changes of other brain regions associated with a change in depressive symptoms and change in cognitive function.

## Materials and methods

### Participants and clinical assessments

This prospective and observational study was approved by the ethics committee of the hospital and performed in accordance with the Declaration of Helsinki. The study was registered at UMIN-CTR (UMIN000019475). Written informed consent was obtained from all participants and/or their surrogate family members. Patients who met the following inclusion criteria were recruited at Komagino Hospital: (1) Diagnostic and Statistical Manual of Mental Disorders, Fourth Edition, Text Revision (DSM-IV-TR) diagnosis of major depressive disorder (MDD), or bipolar (BP) I or II disorder experiencing a major depressive episode (MDE) with melancholic features confirmed by Mini-International Neuropsychiatric Interview; (2) clinical indications for ECT, including pharmacotherapy resistance or requiring a rapid response; (3) age ⩾50 years. The age range was selected based on a previous similar study (Abbott et al., 2014). Exclusion criteria included: (1) any concurrent Axis I diagnosis other than MDD or BP; (2) concurrent drug or alcohol dependence; (3) a diagnosis of neurological or degenerative disorders (e.g. epilepsy, dementia); (4) unstable or severe medical illness (e.g. myocardial infarction within 1 month); (5) pregnancy; (6) ECT treatment within the last 3 months; (7) patients with mandatory admission by the mayor of the municipality. Healthy controls were recruited from the same area. Subjects in this study partly overlap with a previous report (Takamiya et al., 2019a).

Clinical and MRI assessments were conducted within 1 week before the first ECT (TP1) and after 1 week from the last ECT (TP2). Depressive symptoms were evaluated using the 17-item Hamilton Depression Rating Scale (HAM-D), and remission was defined as HAM-D total score ⩽7. The Mini-Mental State Examination (MMSE) was used to assess global cognitive function.

### ECT procedure

Participation in our research did not influence any clinical decision regarding ECT. Informed consent for ECT treatment was obtained independently and prior to recruitment for the study. ECT was provided in clinical settings by each participant's attending psychiatrist. Bilateral ECT was performed using a brief-pulse (0.5 ms) square-wave device (Thymatron system IV device; Somatics, Inc., Lake Bluff, IL, USA). Treatments were performed two to three times a week, in accordance with clinical judgment, and treatments were continued until a plateau was reached and no more improvement was seen in the last two sessions based on clinical assessments. Electroencephalograms (EEG) were recorded to ensure at least 20-s of epileptiform EEG activity after ECT. Propofol (1 mg/kg) was used for general anesthesia, and succinylcholine (0.5–1.0 mg/kg) was used to induce muscle relaxation. Participants continued their psychotropic medications throughout the course of ECT. Incidental use of benzodiazepines or antipsychotics for sleep or anxiety was permitted.

### MRI data acquisition

MRI data were acquired using a 3-Tesla GE Signa HDxt scanner at Komagino Hospital. Whole-brain rs-fMRI scans comprising a total of 188 echo-planar imaging volumes (the first eight volumes were dummy scans) were acquired with the following parameters: TR = 2000 ms, TE = 28 ms, matrix = 64 × 64, slice thickness = 3.5 mm, 36 continuous axial slices, and voxel size = 3.75 × 3.75 × 3.5 mm^3^. During the acquisition, all subjects were instructed to close their eyes and remain awake. Foam padding and earplugs were used to minimize head movement and scanner noise.

High-resolution 3D T1-weighted images were acquired using a fast-spoiled gradient recalled echo sequence (fSPGR: TR = 6.9 ms, TE = 2.9 ms, sagittal orientation, matrix = 256 × 256 mm^2^, slice thickness = 1.0 mm, voxel size = 0.9 × 0.9 × 1.0 mm^3^, 174 slices).

### Functional image preprocessing

All preprocessing was conducted using the CONN Functional Connectivity Toolbox (https://www.nitrc.org/projects/conn) with the SPM12 (www.fil.ion.ucl.ac.uk/spm). After the removal of the first initial 10 scans (eight dummy scans and two functional scans), default-preprocessing pipeline implemented in CONN was used.

The preprocessing pipeline included realignment and unwarping, slice-timing correction, normalization into Montreal Neurological Institute (MNI) space, and smoothing (8-mm Gaussian kernel). To minimize the impact of head motion on estimated FC, the following possible confounders were removed before computing connectivity measures: BOLD signals from white matter (WM) and cerebrospinal fluid (CSF), and motion parameters (realignment and scrubbing parameters). The anatomical component correction (aCompCor) method (Behzadi, Restom, Liau, & Liu, 2007) was used to identify principal components associated with segmented WM and CSF. The Artifact Rejection Toolbox (ART) outlier detection and scrubbing method were used to regress out motion parameters and to eliminate specific frames with motion outliers. The threshold for outliers was set at global-signal *z*-value of 3 and subject-motion of 0.5 mm. We excluded the data from subsequent analysis if more than 36 volumes (i.e. 20% of the acquired volumes) were scrubbed (Wang et al., 2019). The resulting functional images were despiked and band-pass filtered (0.008–0.09 Hz).

### FC analysis

Our analyses included the following two approaches: (1) *a priori* hypothesis testing and (2) data-driven exploratory analysis. Because the effect of ECT on the hippocampus has been consistently reported (Oltedal et al., 2018; Sartorius et al., 2019; Takamiya et al., 2018), we selected bilateral hippocampal seeds (defined by Harvard-Oxford Atlas implemented in CONN) to examine how FCs in these regions were associated with clinical outcomes following ECT. Whole-brain correlation maps were created by calculating Pearson's correlation coefficients between the extracted mean signal time course from the hippocampal seeds and all other voxels. Subject-level seed maps were converted to Fisher-transformed Z scores to allow for second-level general linear model analyses.

Multivoxel pattern analysis (MVPA) is a whole-brain connectomic approach and it is used to replicate hypothesis-driven seed-based analyses, and to conduct whole-brain voxel-wise resting-state FC analysis (Philip et al., 2018; Wang et al., 2019; Whitfield-Gabrieli et al., 2016). For all voxels in each subject and session, MVPA can create the pairwise connectivity pattern between each voxel and the rest of the brain. Principal component analysis (PCA) was used to reduce the dimensionality, and five components were kept to maintain an approximate 5:1 ratio between subjects and number of components (Philip et al., 2018; Whitfield-Gabrieli et al., 2016). Second-level MVPA statistical analysis yields a multivariate pattern of voxel clusters showing connectivity changes associated with clinical improvement after ECT. Because MVPA is an omnibus test, post-hoc analyses are needed to investigate connectivity patterns of each identified cluster. Therefore, the following seed-to-voxel analyses using identified clusters were conducted.

### Statistical analysis

Descriptive statistics were used to describe the study participants. Distributions of all variables were inspected using histograms, q-q plots, and Shapiro-Wilk tests. Because percentage changes in HAM-D were negatively skewed, these variables were squared to obtain a normal distribution. Percentage change in MMSE was positively skewed, and these variables were natural log-transformed to obtain a normal distribution.

To identify the FC change associated with the clinical effect of ECT, the second-level analysis was conducted in the CONN toolbox, including seed-to-voxel maps as seeds, TP1 and TP2 as between-conditions contrast, and percentage change in HAM-D as between-subjects contrast. Age and Sex were included as nuisance covariates. Baseline HAM-D scores were also included as an additional covariate to control for the baseline severity.

For MVPA second-level analyses, MVPA components were included as between-measures contrast, TP1 and TP2 as between-conditions contrast, and percentage change in HAM-D as between-subjects contrast. Age, sex, baseline HAM-D scores were included as nuisance covariates. Then, clusters identified at this step were used as seeds in the following seed-to-voxel analyses.

To investigate the association between ECT-related FC changes and global cognitive changes, all analyses were conducted using percentage change in the MMSE before and after ECT instead of the percentage change in the HAM-D. R ver 3.4.3 was used for statistical analysis except for above-mentioned seed-to-voxel and MVPA analyses. The statistical threshold for voxel-wise whole-brain analyses was set at cluster-level false discovery rate (FDR)-corrected *p* < 0.05 with a voxel height of *p* < 0.001. Mango (http://ric.uthscsa.edu/mango/mango.html) was used to show imaging results.

## Results

### Clinical demographics

Twenty-seven depressed individuals (67.5 ± 8.1 years old; 19 female) participated in the study (Table 1). ECT significantly improved depressive symptoms (HAM-D = 32.0 ± 6.6 at TP1; HAM-D = 6.0 ± 5.3 at TP2) (Paired *t* test: *t* = 16.0, df = 26, *p* < 0.001), and global cognitive function (MMSE = 26.4 ± 3.4 at TP1; MMSE = 27.7 ± 2.4 at TP2) (Paired *t* test: t = 2.49, df = 23, *p* = 0.02). There was no correlation between percentage change in HAM-D scores and percentage change in MMSE scores (*r* = −0.26, df = 21, *p* = 0.24). After ECT, 20 participants (74%) met the remission criteria. All but two participants completed rs-fMRI assessments at both TP1 and TP2.

**Table 1.** Clinical characteristics of participants

	ECT patients
Number	27
Age (years)	67.5 (8.1)
Female, *n* (%)	19 (70.4%)
Bipolar disorder, *n* (%)	5 (18.5%)
Psychotic features, *n* (%)	19 (70.4%)
Family history, *n* (%)	10 (37.0%)
Duration of the current episode (month)	5.0 (3.0–8.5)
Illness duration (year)	5.0 (1.6–15)
Number of ECT	10.8 (1.8)
Charge (mC)	289.2 (85.8)
EEG seizure (seconds)	42.3 (12.8)
Time between TP1 and TP2 (days)	39.1 (7.0)
Baseline HAM-D scores	32.0 (6.6)
HAM-D scores after ECT	6.0 (5.3)
Baseline MMSE scores	26.4 (3.4)
MMSE scores after ECT	27.7 (2.4)
Antidepressants, *n* (%)	24 (88.9%)
Antipsychotics, *n* (%)	21 (77.8%)
Mood stabilizer, *n* (%)	2 (7.4%)
Benzodiazepine, *n* (%)	4 (14.8%)

Each variable is described as mean (s.d.) for continuous variable.

Duration of the current episode and Illness duration are described as median (IQR) because of non-normal distribution.

### Hippocampal seed-to-voxel analysis: correlation with HAM-D changes or MMSE changes

**Fig. 1.**
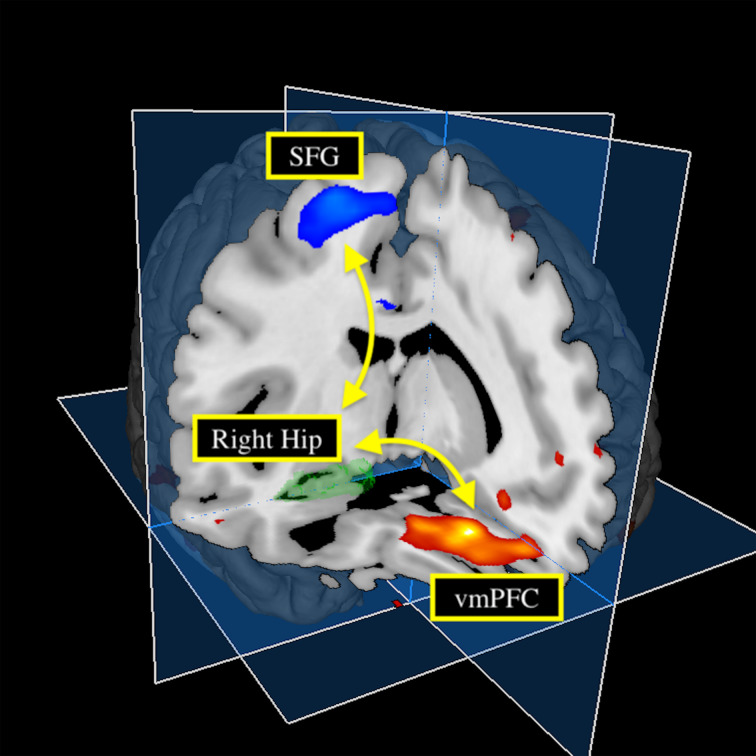
Hippocampal connectivity changes with clinical effect of ECT. Changes in depressive symptom following ECT positively correlated with changes in the right hippocampus-vmPFC connectivity, and negatively correlated with changes in the right hippocampus-SFG connectivity. Hip, hippocampus; vmPFC, ventromedial prefrontal cortex; SFG, superior frontal gyrus.

Connectivity changes between the right hippocampus and one cluster located in the vmPFC, including the SCC, MPFC, and OFC, showed a positive correlation with HAM-D changes (Fig. 1; Table 2). Connectivity changes between the right hippocampus and the right superior frontal gyrus (SFG) showed a negative correlation with HAM-D changes. Connectivity changes of the left hippocampal seed did not show any correlations with HAM-D changes. We investigated the effect of head motion, longitudinal volume change with ECT, medication dosage, and each participant's data on our results, but none of them altered the main results (online Supplementary Material).

**Table 2.** Hippocampal connectivity changes associated with changes in clinical scores

Seed	Brain regions	Peak MNI coordinates			*T* values	*Z* scores	Cluster size (voxels)
		*x*	*y*	*z*			
Right hippocampus	*Positive correlation*						
	Bilateral subcallosal cortex Bilateral frontal medial cortex Right frontal orbital cortex	0	34	−20	6.60	4.76	500
	*Negative correlation*						
	Right superior frontal gyrus	32	−2	58	5.52	4.25	259
Left hippocampus	*Positive correlation*						
	None						
	*Negative correlation*						
	None						

Connectivity changes between the left hippocampus and the frontal pole (FP) showed a positive correlation with MMSE changes, whereas no significant clusters were identified in the right hippocampal seed-to-voxel analysis (online Supplementary Table 1).

### MVPA analysis: correlation with HAM-D changes or MMSE changes

**Fig. 2.**
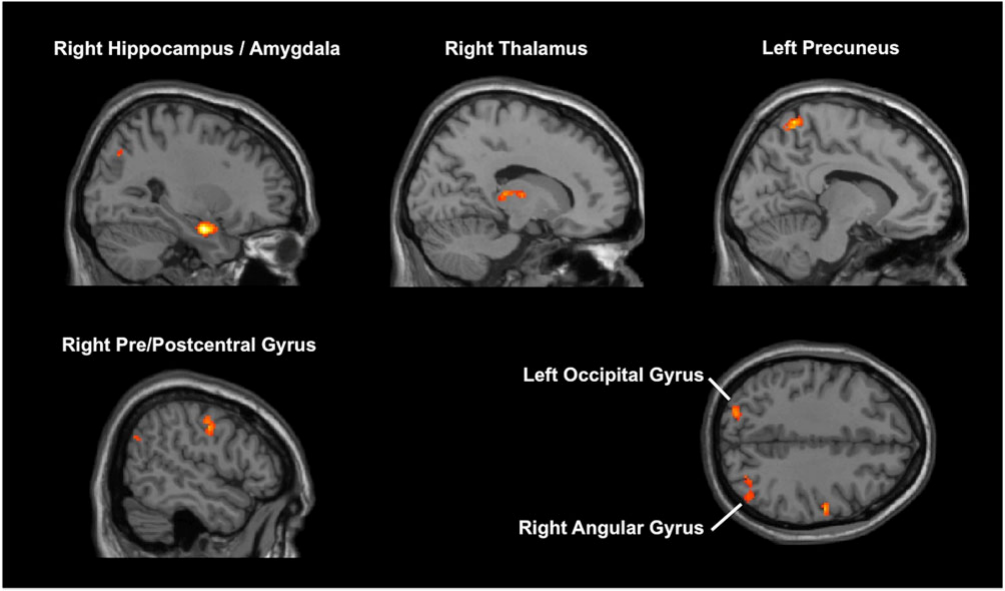
Results of multi-voxel pattern analysis (MVPA). MVPA identified six brain regions associated with improvement in depressive symptoms.

MVPA identified six clusters associated with HAM-D changes (online Supplementary Table 2; Fig. 2). One of these clusters was the right medial temporal lobe (MTL), including the hippocampus. The following seed-to-voxel analysis using the MVPA-defined right MTL cluster replicated our hypothesis-driven right hippocampal seed-to-voxel analysis (online Supplementary Table 3).

Results of the other MVPA-identified seeds-to-voxel analyses are described in online Supplementary Table 3. In short, HAM-D changes showed a positive correlation with the following FC changes: (1) right MTL and several other brain regions, including SFG, MPFC, SCC, and precuneus; (2) bilateral precuneus and left occipital cortex. HAM-D changes showed a negative correlation with the following FC changes: (1) left precuneus and left MPFC; (2) right thalamus and left pre/postcentral gyrus; (3) right pre/postcentral gyrus and anterior cingulate gyrus.

MVPA identified two clusters (e.g. brainstem and left precentral gyrus) associated with MMSE changes (online Supplementary Table 2). The results of MVPA did not include the left hippocampus but results of the MVPA-identified seeds-to-voxel analyses included the left hippocampus and left FP (online Supplementary Table 4).

## Discussion

The current results show that depressive symptom improvement after ECT was associated with right hippocampal FC changes. MVPA analysis revealed that depressive symptom improvement was associated with FC changes among the MTL, thalamus, sensorimotor cortex, and precuneus. Because ECT is the most effective and rapid antidepressant treatment, understanding of the neuronal networks associated with ECT-related antidepressant effects may lead to the development of precisely targeted antidepressant treatments.

Both hypothesis-driven seed-based analyses and data-driven whole-brain analyses revealed that ECT-induced increased FC between MTL, including the hippocampus, and the vmPFC was associated with depressive symptom improvement. The vmPFC plays a critical role in both positive and negative emotional processing (Myers-Schulz & Koenigs, 2012). Functional imaging studies have shown that the vmPFC was hyperactive during a depressed state and that recovery from depression was associated with decreased activity in the vmPFC (Koenigs & Grafman, 2009). Direct stimulation of the SCC, a part of the vmPFC, demonstrated an antidepressant effect in treatment-resistant depression (Mayberg et al., 2005). Recent studies emphasized that the antidepressant effect of SCC-DBS was mediated by modulation of certain neuronal networks rather than modulation of SCC itself (Choi, Riva-Posse, Gross, & Mayberg, 2015; Riva-Posse et al., 2018). These studies reported that stimulation of the uncinate fasciculus, which connects vmPFC and MTL, was one of the critical pathways for mediating rapid-onset relief of depression (Choi et al., 2015) and achieving a long-term antidepressant effect (Riva-Posse et al., 2018). The uncinate fasciculus includes bidirectional monosynaptic fibers connecting vmPFC and MTL (Catani, Howard, Pajevic, & Jones, 2002), and abnormal structural connectivity among these regions was reported in depression (Dalby et al., 2010; Zhang et al., 2012). A recent large neuroimaging study revealed that resting-state FC between vmPFC and MTL was abnormally reduced in depression (Cheng et al., 2016). Given all this evidence, our results indicate that the modulation of the frontotemporal connectivity, especially the right hippocampus-vmPFC connectivity, may mediate the potent and rapid antidepressant effects of ECT.

MVPA analysis showed that FC change in the thalamus was associated with depressive symptom improvement. The thalamus is a central hub of the cortico-striatal-thalamic-cortical (CSTC) loop, and it plays a critical role in relaying information from peripheral organs to sensory cortices as well as in cortico–cortical interactions (Sherman & Guillery, 2017). Structural abnormalities in the thalamus have been shown in late-life depression (Bora, Harrison, Davey, Yücel, & Pantelis, 2012). Abnormalities in thalamic FC have been identified in depression, including increased thalamic connectivity with SCC (Greicius et al., 2007), increased thalamocortical connectivity (Cheng et al., 2016), and increased thalamo-temporal as well as thalamo-somatosensory connectivity (Brown, Clark, Hassel, MacQueen, & Ramasubbu, 2017). The thalamus is a critical region for seizure propagation (Blumenfeld et al., 2009; Enev et al., 2007), as well as an important region for the underlying mechanisms of ECT action (Farzan, Boutros, Blumberger, & Daskalakis, 2014). Pretreatment smaller thalamic volume and resting-state activity predicted a worse outcome for ECT (Takamiya et al., 2019c). Longitudinal changes in the thalamic FC were associated with clinical improvement in depression (Leaver et al., 2016), and longitudinal cerebral blood flow change in the thalamus and somatosensory cortex were observed only in ECT responders (Leaver et al., 2019). A previous positron emission tomography study also reported increased blood flow during and following ECT (Takano et al., 2007). Moreover, thalamic FC modulation may be specific to ECT. Previous studies, which utilized MVPA to investigate FC changes associated with improvement of depression before and after TMS or antidepressant medications, reported similar significant brain regions (e.g. right MFG, ACC, and insula), which were also identified in the current study. However, unlike our study, neither of the previous TMS nor antidepressant studies reported the thalamus as a significant brain region (online Supplementary Table 6). The current results support previous findings for the importance of the thalamus as the mechanisms of ECT action.

MVPA analysis also showed that FC change in the precuneus was associated with depressive improvement. The precuneus is a central hub of the posterior default mode network (DMN), which is implicated in self-referential processing (Sheline et al., 2009). A previous meta-analysis revealed that depressed patients showed increased FC within DMN nodes (Kaiser et al., 2015). The effect of ECT on DMN has been reported in previous fMRI studies (Abbott et al., 2013; Mulders et al., 2016; Wang et al., 2018), although the clinical relevance of these effects was unclear. The current result of the association between clinical improvement and decreased FC within DMN nodes, specifically between posterior DMN and anterior DMN (e.g. decreased precuneus-MPFC connectivity), adds to our understanding of the effect of ECT on DMN.

Some limitations should be acknowledged. First, the sample size was modest, although comparable to or larger than previous ECT imaging studies. Patients who need ECT are the most severe cases (e.g. baseline mean HAM-D score was 32.0 in the study, and most of them had psychotic features) and it may be difficult for them to participate in longitudinal MRI studies. In the current study, however, we confirmed our results by investigating the effect of each individual's data on the results (online Supplementary Material). Second, our cohort continued taking medications, which may affect the results of FC changes. Discontinuation of medications was not ethical given the severity of our cohort. We confirmed that our main results did not change after including medication dosage as covariates in multiple regression analyses (online Supplementary Material). Third, because our cohort includes only elderly depressed patients, it is difficult to generalize our results to non-geriatric patients. In contrast, our cohort includes both patients with MDD and BP, because we focused on longitudinal FC changes associated with improvements in severe melancholic depressive symptoms with ECT based on a dimensional approach. Indeed, there is an argument that identifying the shared neurobiology underlying common symptoms across disease categories may be suitable for personalized treatments (Insel et al., 2010; Nusslock & Alloy, 2017). However, because there is a possibility that a categorical diagnosis may affect the longitudinal effects of ECT on the brain, a future study including a larger cohort is warranted to address this argument. Fourth, MMSE includes multiple cognitive domains, and may not detect subtle cognitive changes induced by ECT (Moirand et al., 2018). Therefore, our analysis regarding cognitive change was exploratory, and the results regarding cognitive changes should not be overemphasized. Future studies should focus on change in specific cognitive domains that relate to ECT.

## Conclusions

In conclusion, depressive symptom improvement after ECT was associated with a reorganization of the neuronal network, including the hippocampus, thalamus, sensorimotor cortex, and precuneus. Modulation of these networks may be the underlying mechanisms of ECT action. Our results have important implications for developing precisely targeted neuromodulation treatments.

## Supplementary material

For supplementary material accompanying this paper visit https://doi.org/10.1017/S0033291720001518. click here to view supplementary material
