# Supplementary material for: Neuronal network mechanisms associated with depressive symptom improvement following electroconvulsive therapy
Source: Psychol Med. 2020 Jun 1;51(16):2856–63. doi: 10.1017/S0033291720001518 (PMC8640363; doi:10.1017/S0033291720001518)
Supplement: Supplementary file 1 [file S0033291720001518sup001.docx]

*Supplementary material*

Supplementary Figure 1. Scatter plots of changes in the hippocampal connectivity and changes in clinical scores.


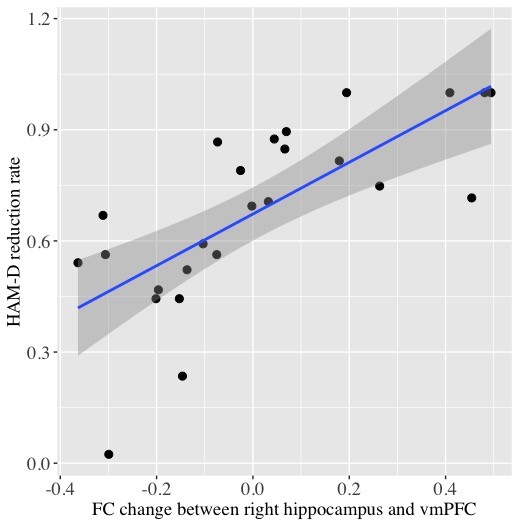


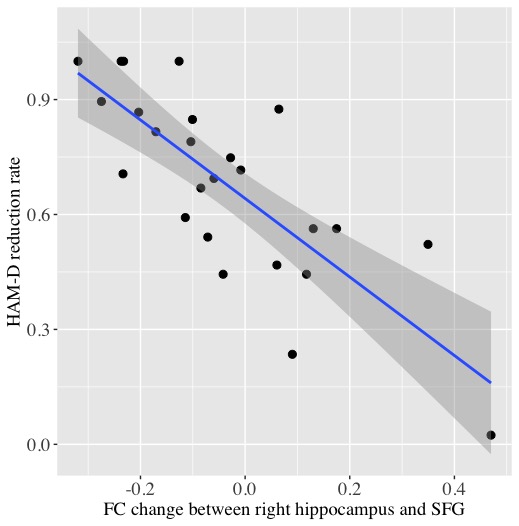


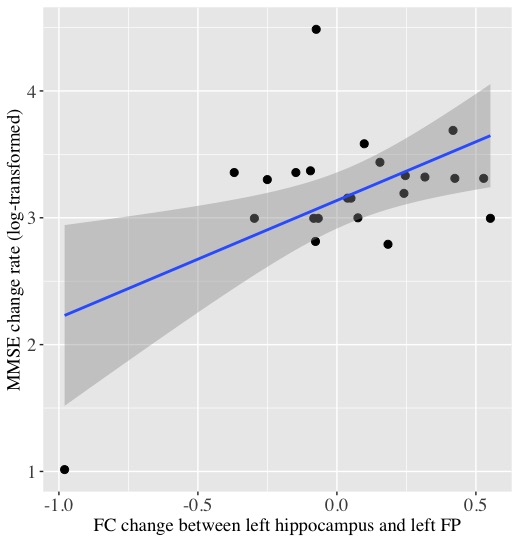


FC, functional connectivity; vmPFC, ventromedial prefrontal cortex; SFG, superior frontal gyrus; FP, frontal pole

Supplementary Table 1. Hippocampal connectivity changes associated with cognitive change

| Seed | Brain Regions | Peak MNI coordinates | | | T values | Z scores | Cluster Size |
| --- | --- | --- | --- | --- | --- | --- | --- |
|  |  | x | y | z |  |  | (voxels) |
| Right Hippocampus | *Positive correlation* |  |  |  |  |  |  |
|  | None |  |  |  |  |  |  |
|  | *Negative correlation* |  |  |  |  |  |  |
|  | None |  |  |  |  |  |  |
|  |  |  |  |  |  |  |  |
| Left Hippocampus | *Positive correlation* |  |  |  |  |  |  |
|  | Bilateral Frontal Pole | 0 | 62 | −14 | 5.74 | 4.27 | 233 |
|  | *Negative correlation* |  |  |  |  |  |  |
|  | None |  |  |  |  |  |  |

*Multivoxel pattern analysis (MVPA)*

MVPA is a well-validated whole brain voxel-wise functional connectivity analysis. MVPA calculates functional connectivity between one voxel and the rest of the brain, and it repeats this process across entire brain. Principal component analysis (PCA) was used to reduce the dimensionality, and five components were kept to maintain an approximate 5:1 ratio between subjects and number of components (Philip et al., 2018; Hair et al., 1998). Second-level MVPA statistical analysis yields a multivariate pattern of voxel clusters showing connectivity changes associated with change in clinical score after ECT. Because MVPA is an omnibus test, post-hoc analyses are needed to investigate connectivity patterns of each identified cluster. Clusters identified by MVPA are listed in Supplementary Table 2. The results of the seed-to-voxel analyses using MVPA-derived clusters were described in Supplementary Table 3 (MVPA: correlation with symptom improvement) and Supplementary Table 4 (MVPA: correlation with cognitive change).

Supplementary Table2. Clusters associated with clinical scores identified by multivoxel pattern analysis

| Clinical score | Brain Regions | Peak MNI coordinates | | | F values | Z scores | Cluster Size (voxels) |
| --- | --- | --- | --- | --- | --- | --- | --- |
|  |  | x | y | z |  |  |  |
| HAM-D | Right Hippocampus/Amygdala | 28 | −4 | −20 | 17.5 | 4.50 | 140 |
|  | Left Precuneus | −10 | −54 | 68 | 11.4 | 3.67 | 123 |
|  | Right Thalamus | 18 | −28 | 8 | 10.4 | 3.56 | 107 |
|  | Right Angular Gyrus | 46 | −68 | 34 | 6.7 | 2.74 | 100 |
|  | Left Occipital Cortex | −24 | −82 | 42 | 10.7 | 3.56 | 89 |
|  | Right Pre/Postcentral Gyrus | 54 | −8 | 38 | 9.6 | 3.36 | 86 |
|  |  |  |  |  |  |  |  |
| MMSE | Brainstem | −4 | −44 | −44 | 16.3 | 4.28 | 126 |
|  | Left Precentral Gyrus | −16 | −20 | 74 | 15.6 | 4.21 | 122 |

Supplementary Table 3. Connectivity changes with MVPA-derived seeds associated with symptom improvement

| MVPA-derived Seeds | Brain Regions | Peak MNI coordinates | | | T values | Z scores | Cluster Size (voxels) |
| --- | --- | --- | --- | --- | --- | --- | --- |
|  |  | x | y | z |  |  |  |
| Right Hippocampus/Amygdala |  |  |  |  |  |  |  |
|  | *Positive correlation* |  |  |  |  |  |  |
|  | Left Superior Frontal Gyrus | −12 | 32 | 62 | 6.90 | 4.88 | 225 |
|  | Left Middle Frontal Gyrus | −40 | 14 | 50 | 6.81 | 4.84 | 252 |
|  | Bilateral Frontal Medial Cortex Bilateral Subcallosal Cortex Bilateral Frontal Pole | 2 | 50 | −22 | 6.35 | 4.65 | 231 |
|  | Left Angular Gyrus Left Inferior Parietal Lobule | −40 | −66 | 38 | 5.58 | 4.28 | 664 |
|  | Right Middle Frontal Gyrus | 48 | 24 | 44 | 4.78 | 3.86 | 135 |
|  | Bilateral Precuneus Bilateral Posterior Cingulate Gyrus | −2 | −46 | 36 | 4.45 | 3.67 | 162 |
|  | *Negative correlation* |  |  |  |  |  |  |
|  | Left Planum Temporale Left Parietal Operculum Cortex | −56 | −32 | 12 | 6.29 | 4.62 | 386 |
|  |  |  |  |  |  |  |  |
| Left precuneus |  |  |  |  |  |  |  |
|  | *Positive correlation* |  |  |  |  |  |  |
|  | None |  |  |  |  |  | 2 |
|  | *Negative correlation* |  |  |  |  |  |  |
|  | Bilateral Frontal Pole | 8 | 54 | 8 | 5.95 | 4.46 | 144 |
|  | Left Frontal Medial Cortex | −4 | 46 | −20 | 5.40 | 4.19 | 178 |
|  | Left Superior Frontal Gyrus | −2 | 48 | 36 | 5.39 | 4.19 | 268 |
|  | Left Inferior Frontal Gyrus | −54 | 24 | 10 | 5.16 | 4.07 | 144 |
|  |  |  |  |  |  |  |  |
| Right Thalamus |  |  |  |  |  |  |  |
|  | *Positive correlation* |  |  |  |  |  |  |
|  | None |  |  |  |  |  |  |
|  | *Negative correlation* |  |  |  |  |  |  |
|  | Left Pre/Postcentral Gyrus | −8 | −34 | 66 | 5.93 | 4.45 | 185 |
|  |  |  |  |  |  |  |  |
| Left Occipital Cortex |  |  |  |  |  |  |  |
|  | *Positive correlation* |  |  |  |  |  |  |
|  | Bilateral Precuneus | −12 | −60 | 24 | 5.51 | 4.25 | 260 |
|  | Left Superior Frontal Gyrus | −18 | 34 | 58 | 4.71 | 3.82 | 415 |
|  | *Negative correlation* |  |  |  |  |  |  |
|  | None |  |  |  |  |  |  |
|  |  |  |  |  |  |  |  |
| Right Pre/Postcentral Gyrus |  |  |  |  |  |  |  |
|  | *Positive correlation* |  |  |  |  |  |  |
|  | None |  |  |  |  |  |  |
|  | *Negative correlation* |  |  |  |  |  |  |
|  | Bilateral Anterior Cingulate Gyrus | 8 | 50 | 12 | 6.82 | 4.85 | 647 |
|  | Left Superior Frontal Gyrus | −14 | 28 | 48 | 5.41 | 4.20 | 288 |

Supplementary Table 4. Connectivity changes with MVPA-derived seeds associated with cognitive change

| MVPA-derived Seeds | Brain Regions | Peak MNI coordinates | | | T values | Z scores | Cluster Size (voxels) |
| --- | --- | --- | --- | --- | --- | --- | --- |
|  |  | x | y | z |  |  |  |
| Brainstem |  |  |  |  |  |  |  |
|  | *Positive correlation* |  |  |  |  |  |  |
|  | Anterior Cingulate Gyrus | −2 | 10 | 18 | 6.70 | 4.69 | 302 |
|  | Left Temporal Pole | −50 | −10 | −28 | 5.38 | 4.10 | 293 |
|  | Left Frontal Pole | −50 | 46 | −8 | 6.64 | 4.66 | 191 |
|  | *Negative correlation* |  |  |  |  |  |  |
|  | Right Superior Parietal Lobule | 18 | −50 | 74 | 7.37 | 4.94 | 721 |
|  | Right Supramarginal Gyrus | 62 | −34 | 38 | 6.99 | 4.80 | 249 |
|  | Right Superior Parietal Lobule | 38 | −42 | 42 | 5.29 | 4.06 | 180 |
|  | Left Supramarginal Gyrus | −58 | −40 | 42 | 5.01 | 3.91 | 160 |
| Left Precentral Gyrus |  |  |  |  |  |  |  |
|  | *Positive correlation* |  |  |  |  |  |  |
|  | left Frontal Pole | −12 | 46 | 38 | 6.97 | 4.79 | 650 |
|  | Left Hippocampus Brainstem | −16 | −18 | −18 | 5.21 | 4.02 | 297 |
|  | Anterior Cingulate Gyrus | 0 | 8 | 34 | 6.71 | 4.69 | 217 |
|  | *Negative correlation* |  |  |  |  |  |  |
|  | Right Superior Parietal Lobule | 30 | −58 | 66 | 5.15 | 3.99 | 535 |
|  | Right Lateral Occipital Cortex | 14 | −74 | 56 | 5.76 | 4.28 | 242 |

*Effect of head motion*

We investigated the effect of head micromovements on our main results by calculating mean frame wise displacement (FD) in the CONN toolbox. There were no significant differences in mean FD values between TP1 (average = 0.16, s.d. = 0.060) and TP2 (average = 0.16, s.d. = 0.050) (Paired t-test: t = −0.13, df =24, p = 0.90). In multiple regression models, including age, sex, baseline HAM-D scores, and changes in mean FD as covariates, both right hippocampus-vmPFC connectivity changes and right hippocampus-SFG connectivity changes predicted percentage change in HAM-D scores (all ps <0.001).

In the current study, the scrubbing method was used to eliminate specific frames with motion outliers. The threshold for outliers was set at global-signal z-value of 3 and subject-motion of 0.5 mm. All data did not meet the pre-defined exclusion criteria (i.e., censored more than 20% of the acquired volumes) (Supplementary Table 5). There were no significant changes in the number of detected outliers between TP1 and TP2 (p = 0.10). In multiple regression models, including age, sex, baseline HAM-D scores, and changes in the number or percentage of censored volumes as covariates, both right hippocampus-vmPFC connectivity changes and right hippocampus-SFG connectivity changes predicted percentage change in HAM-D scores (all ps <0.001). Moreover, in multiple regression models, including age, sex, baseline MMSE scores, and changes in mean FD or changes in censored volumes as covariates, left hippocampus-FP connectivity changes predicted percentage change in MMSE (all ps = 0.001).

Supplementary Table 5. Censored volumes per subject

| Subject | TP1, #volumes | TP1, %volumes | TP2, #volumes | TP2, %volumes |
| --- | --- | --- | --- | --- |
| 1 | 19 | 10.67 | 11 | 6.18 |
| 2 | 4 | 2.25 | 0 | 0.00 |
| 3 | 0 | 0.00 | 0 | 0.00 |
| 4 | 10 | 5.62 | 8 | 4.49 |
| 5 | 0 | 0.00 | 2 | 1.12 |
| 6 | 0 | 0.00 | 0 | 0.00 |
| 7 | 2 | 1.12 | 8 | 4.49 |
| 8 | 2 | 1.12 | 0 | 0.00 |
| 9 | 17 | 9.55 | 4 | 2.25 |
| 10 | 17 | 9.55 | 0 | 0.00 |
| 11 | 4 | 2.25 | 4 | 2.25 |
| 12 | 8 | 4.49 | 12 | 6.74 |
| 13 | 0 | 0.00 | 0 | 0.00 |
| 14 | 14 | 7.87 | 0 | 0.00 |
| 15 | 0 | 0.00 | 16 | 8.99 |
| 16 | 16 | 8.99 | 12 | 6.74 |
| 17 | 8 | 4.49 | 0 | 0.00 |
| 18 | 7 | 3.93 | 11 | 6.18 |
| 19 | 2 | 1.12 | 2 | 1.12 |
| 20 | 0 | 0.00 | 4 | 2.25 |
| 21 | 12 | 6.74 | 4 | 2.25 |
| 22 | 12 | 6.74 | 0 | 0.00 |
| 23 | 2 | 1.12 | 0 | 0.00 |
| 24 | 2 | 1.12 | 2 | 1.12 |
| 25 | 8 | 4.49 | 4 | 2.25 |

*Effect of GMV change*

Because ECT-induced hippocampal volume increase has been reported consistently (Takamiya et al., 2018), we investigated the effect of longitudinal changes in hippocampal GMV on FC analyses in the current study. We calculated GMV in the hippocampal seed region using the SPM12, and we replicated ECT-induced hippocampal volume increase by using paired t-tests (right hippocampus: t = 5.43, df = 24, p <0.001; left hippocampus: t = 4.57, df = 24, p <0.001).

Right hippocampal GMV change did not correlate with right hippocampus-vmPFC connectivity change (r = 0.27, df = 23, p = 0.19) nor right hippocampus-SFG connectivity change (r = −0.34, df = 23, p = 0.09). In multiple regression models, including age, sex, baseline HAM-D scores, and GMV changes as covariates, our main results did not change: both right hippocampus-vmPFC connectivity changes and right hippocampus-SFG connectivity changes predicted percentage change in HAM-D (all ps <0.001).

Left hippocampal GMV change did not correlate with left hippocampus-FP connectivity change (r = 0.07, df = 21, p = 0.74). In multiple regression models, including age, sex, baseline MMSE scores, and GMV changes in the left hippocampus as covariates, our main results did not change: the left hippocampus-FP connectivity changes predicted percentage change in HAM-D (p = 0.001).

*Effect of medication dosage on FC measurements*

Medication dosage was calculated using defined daily dose (DDD) (<http://www.whocc.no/atc_ddd_index/>). DDD is defined as an assumed average maintenance dose per day for a drug used for its main indication in adults. In multiple regression analyses, including age, sex, baseline HAM-D scores, and DDDs of each medication, the main results did not change: both hippocampus-vmPFC connectivity changes and hippocampus-SFG connectivity changes predicted percentage changes in HAM-D scores (all ps <0.001). Moreover, in multiple regression analyses, including age, sex, baseline MMSE scores, and DDDs of each medication, the main results did not change: left hippocampus-FP connectivity changes predicted percentage changes in MMSE scores (p = 0.002).

*Effect of each participant’s data: Leave-one-out cross-validation*

We use leave-one-sample-out cross-validation (LOOCV) to test cluster validity, and to investigate the effect of each participant's data, as a previous similar study conducted (Philip et al., 2018). In this method, group-level generalized linear models were re-estimated using N-1 participants. As a result, vmPFC was identified 100% cross-validated, and SFG was identified 84% cross-validated. Because both clusters were identified more than 80% threshold (Philip et al., 2018), we reported both clusters in the main text. As a result of LOOCV for analysis regarding cognitive change, the left FP cluster was identified 91.3 % cross validated.

*Overview of previous similar research*

Previous research utilized MVPA, which is implemented in the CONN toolbox, to investigate changes in functional connectivity associated with depressive symptom improvement via antidepressant treatments, including transcranial magnetic stimulation (TMS) and pharmacotherapy (Supplementary Table 6). There were common identified regions (e.g., right middle frontal gyrus, insula, anterior cingulate cortex, and somatosensory cortex) between these studies, some of which were also identified in our study. In contrast, the thalamus and occipital cortex, which were identified in the current study, may be specific to the effect of ECT. However, this interpretation should be acknowledged with caution, because there were differences in the assessment of depressive severity and participants’ characteristics (e.g., age distribution) between our study and previous studies.

Supplementary Table 6. Overview of previous research utilizing MVPA

|  | Philips et al., 2018 | Wang et al., 2019 |
| --- | --- | --- |
| Treatment | TMS | Medication (SNRI) |
| Assessment | IDSSR | HAM-D |
| Number | 25 | 31 (active treatment in RCT) |
| Age, mean (SD) | 51.3 (11.1) | 38.8 (12.6) |
| MVPA | right MFG, right insula, left ACC, left postcentral gyrus, right fusiform gyrus | right MFG, right FP, insula, ACC,  Precentral gyrus, SMA, left secondary somatosensory cortex, right fusiform gyrus, STG, right TP, PCC, left SPL, SMG, periaqueductal gray, right cerebellum |

MVPA, multivoxel pattern analysis; TMS, transcranial magnetic stimulation; SNRI, serotonin and norepinephrine reuptake inhibitor; IDSSR, Inventory of Depressive Symptoms-Self Report; HAM-D, Hamilton Depression Rating Scale; MFG, middle frontal gyrus; ACC, anterior cingulate cortex; FP, frontal pole; SMA, supplementary motor area; STG, superior temporal gyrus; TP, temporal pole; PCC, posterior cingulate gyrus; SPL, superior parietal lobule; SMG, supramarginal gyrus

*References*

Hair, J., Anderson, R., Tatham, R., Black, W. (1998). Multivariate Data Analysis, 5th ed. Englewood Cliffs, NJ: Prentice-Hall.

Philip, N. S., Barredo, J., van ’t Wout-Frank, M., Tyrka, A. R., Price, L. H., & Carpenter, L. L. (2018). Network Mechanisms of Clinical Response to Transcranial Magnetic Stimulation in Posttraumatic Stress Disorder and Major Depressive Disorder. *Biological Psychiatry*, *83*(3), 263–272.

Takamiya, A., Chung, J. K., Liang, K. C., Graff-Guerrero, A., Mimura, M., & Kishimoto, T. (2018). Effect of electroconvulsive therapy on hippocampal and amygdala volumes: Systematic review and meta-analysis. *British Journal of Psychiatry*, 212(1), 19–26.

Wang, Y., Bernanke, J., Peterson, B.S., McGrath, P., Stewart, J., Chen, Y., … Posner, J. (2019). The association between antidepressant treatment and brain connectivity in two double-blind, placebo-controlled clinical trials: a treatment mechanism study. *The Lancet Psychiatry* 6(8), 667–674.
